# Managing Modern Antiretroviral Therapy in the Intensive Care Unit: Overcoming Challenges for Critically Ill People With Human Immunodeficiency Virus

**DOI:** 10.1093/ofid/ofae213

**Published:** 2024-04-17

**Authors:** Daniel B Chastain, Patrick J Tu, Marisa Brizzi, Chelsea A Keedy, Aubrey N Baker, Brittany T Jackson, Amber F Ladak, Leslie A Hamilton, Nicholas R Sells, Andrés F Henao-Martínez, Kathleen A McManus, David B Cluck

**Affiliations:** Department of Clinical and Administrative Pharmacy, University of Georgia College of Pharmacy, Albany, Georgia, USA; Department of Pharmacy, Charlie Norwood Veterans Affairs Medical Center, Augusta, Georgia, USA; Department of Pharmacy, University of Cincinnati Medical Center, Cincinnati, Ohio, USA; Department of Clinical and Administrative Pharmacy, University of Georgia College of Pharmacy, Savannah, Georgia, USA; Department of Clinical and Administrative Pharmacy, University of Georgia College of Pharmacy, Albany, Georgia, USA; Department of Pharmacy, Mount Sinai Morningside, New York, New York, USA; Division of Infectious Diseases, Augusta University, Augusta, Georgia, USA; College of Pharmacy, University of Tennessee Health Science Center, Knoxville, Tennessee, USA; Division of Infectious Diseases, Mount Sinai Morningside, New York, New York, USA; Division of Infectious Diseases, University of Colorado, Aurora, Colorado, USA; Division of Infectious Diseases and International Health, University of Virginia School of Medicine, Charlottesville, Virginia, USA; Department of Pharmacy, University of Virginia Health, Charlottesville, Virginia, USA

**Keywords:** antiretroviral agents, critical care, HIV infections, HIV integrase inhibitors, intensive care units

## Abstract

People with human immunodeficiency virus (HIV) have a 50% excess risk for intensive care unit (ICU) admission, often for non-HIV-related conditions. Despite this, clear guidance for managing antiretroviral therapy (ART) in this setting is lacking. Selecting appropriate ART in the ICU is complex due to drug interactions, absorption issues, and dosing adjustments. Continuing ART in the ICU can be challenging due to organ dysfunction, drug interactions, and formulary limitations. However, with careful consideration, continuation is often feasible through dose adjustments or alternative administration methods. Temporary discontinuation of ART may be beneficial depending on the clinical scenario. Clinicians should actively seek resources and support to mitigate adverse events and drug interactions in critically ill people with HIV. Navigating challenges in the ICU can optimize ART and improve care and outcomes for critically ill people with HIV. This review aims to identify strategies for addressing the challenges associated with the use of modern ART in the ICU.

## Teaser

The absence of clear guidance on the use of antiretroviral therapy (ART) in critical care settings interferes with optimal care for individuals with HIV requiring intensive care unit (ICU) admission. This review offers strategies for overcoming challenges associated with modern ART in the ICU.

Despite advances in antiretroviral therapy (ART), people with human immunodeficiency virus (PWH) remain a vulnerable and high-risk patient population for admission to the hospital [1]. The rate of hospitalization among PWH has decreased from 20–30 [2] to around 13 [3, 4] admissions per 100 individuals annually in recent years [4, 5]. Those who require hospitalization are older and are more likely to have chronic comorbidities and higher CD4 counts and to be virologically suppressed [4]. However, PWH have a 50% excess risk for admission to the intensive care unit (ICU), which may be increasing [6, 7]. The high rates of admission to the ICU observed in PWH often place them at a repeated risk for pharmacotherapy-related complications and errors [1, 8].

The widespread use of ART has dramatically reduced the incidence of opportunistic infections (OIs) [9]. Consequently, this has shifted the primary causes of ICU admissions for PWH, with a notable increase in non–human immunodeficiency virus (HIV)–related conditions and complications arising from other comorbidities, especially cardiovascular and chronic respiratory diseases [1, 6, 7]. This continues to become more prevalent as larger populations of PWH who have been well controlled on ART continue to age and develop other comorbid conditions, often at an earlier age than their counterparts without HIV [10, 11]. Unfortunately, in populations where access to ART is not well established, a significant proportion of ICU admissions remain attributed to OIs, primarily *Pneumocystis jirovecii* pneumonia [12].

Ideally, all hospital admissions for PWH should adhere to established standards of care, including a comprehensive medication reconciliation and history upon admission or presentation to the emergency department [13]. For hospital visits that result in ICU admission, medication-related considerations and nuances should be carefully reevaluated at each care transition throughout the hospitalization. Although it may seem like a straightforward process, challenges arise due to inadequate knowledge of antiretrovirals, the similarity in their names, and the absence or limited availability of prescription data in electronic medical records linked with pharmacy claims [14]. This is further complicated by the critical and complex presentation of PWH requiring ICU admission, making it difficult to promptly obtain detailed and accurate medication histories. Moreover, the most vulnerable PWH may not be receiving appropriate ART, while others may struggle or are unable to provide the information necessary for a thorough medication history. Each care transition leading to an ICU admission introduces a new and cumulative risk of medication errors related to ART, as well as medications used for the prevention and treatment of OIs. Most errors in PWH occur on admission and are propagated throughout the admission, with more than one-third remaining unresolved at the time of discharge [13].

Clinicians must maintain an updated baseline knowledge of these medications to remain aware of considerations surrounding drug interactions, potential medication errors, route of administration considerations, and parameters for holding ART and prophylactic medications. Previous reviews have been published [15–19], but none have included data after January 2021 [19]. Furthermore, none of these reviews have focused on modern ART, which includes integrase strand transfer inhibitors (INSTIs), newer generations of nonnucleoside reverse transcriptase inhibitors (NNRTIs) and protease inhibitors, capsid inhibitors, single-tablet regimens (STRs), and long-acting injectable (LAI) formulations containing multiple antiretrovirals. These modern ART options have gained popularity due to their increased usage, improved adherence, tolerability, potency, and durability compared to older antiretrovirals [20]. This narrative review aims to provide insights into overcoming challenges with the use of modern ART in critically ill patients with HIV requiring ICU admission.

## INITIATING ART IN PWH WHO ARE CRITICALLY ILL

Earlier initiation of ART in PWH leads to significant reductions in morbidity and mortality [21]. Therefore, ART is recommended for all PWH as soon as possible after initial diagnosis to prevent disease progression and transmission. However, the timing of ART initiation for PWH with certain OIs varies [22].

For most OIs, ART should be started within 2 weeks, as early treatment reduces AIDS-related deaths by 50% without increasing adverse events or immune reconstitution inflammatory syndrome (IRIS) [21, 23]. However, there are specific exceptions. Notably, ART initiation should be delayed for 4 to 6 weeks after starting antifungal treatment for cryptococcal meningoencephalitis [24], for 4 to 6 weeks in coccidioidomycosis, especially in those with CD4 cell counts <250 cells/μL or meningitis [22], and between 2 and 8 weeks after starting antituberculous therapy, depending on the CD4 count [22]. These delays are recommended due to the increased risk of IRIS and the potential for unfavorable outcomes in these specific cases.

Early ART initiation for PWH offers substantial benefits such as improved linkage to care, faster viral load suppression, and reduction of both mortality and OIs in clinic-based studies [21, 23]. Immediate ART initiation on the same day or within 72 hours of diagnosis is strongly recommended, but delays in confirmatory testing, ensuring robust and continuous medication access, evaluation for OIs, and concerns about adherence can significantly postpone ART initiation in patients who are newly diagnosed. While rapid ART initiation is much more feasible in the ambulatory setting, few patients who are newly diagnosed with HIV are started on ART prior to hospital discharge, highlighting the need for early ART initiation in hospital settings [24]. However, the lack of recent data hampers our understanding of current trends with modern ART. Additionally, missed opportunities to start ART in PWH receiving care in the ICU are likely, as their prevalence is unknown. Before starting ART in PWH, including those in the ICU, a comprehensive assessment of clinical, behavioral, and social factors, including comorbidities, medication interactions, substance use, medication access, adherence concerns, and housing instability, should be assessed. However, assessing these factors can be particularly complex when patients are intubated, sedated, or recovering from trauma. Continuous ART access and postdischarge linkage to care are essential for optimal outcomes. While adherence concerns by clinicians often lead to delays [25], research indicates limited accuracy in predicting individual adherence [26]. Strategies focusing on addressing underlying contributors to potential nonadherence, such as medication access, mental health concerns, substance use, food insecurity, unstable housing or living conditions, perceived or anticipated stigma, and medical mistrust, are more productive than delaying ART based on predicted nonadherence.

A randomized, open-label clinical trial conducted in Brazil between January 2012 and December 2015 compared outcomes between PWH who were initiated on ART within 5 days of ICU admission (n = 57) and those started on ART after ICU discharge (n = 58) [27]. No difference in mortality was observed throughout hospitalization (67% vs 64%, *P* = .75) or at 6-month follow-up (68% vs 79%, *P* = .20), though the study was prematurely discontinued due to slow recruitment and inability to enroll enough participants to meet power. Baseline characteristics were similar, but large proportions of patients in both groups had OIs, including tuberculosis, cryptococcosis, and toxoplasmosis. Among patients in the early start group, 4 died prior to starting ART while 7 started ART 5 days after ICU admission. Alternatively, 64% of patients in the late start group did not initiate ART during hospitalization. Adverse events attributed to ART occurred in 10% of patients in both groups. In addition, details regarding the selection and availability of ART in Brazil during the study period, follow-up process, and postdischarge support were not provided. Although this study did not demonstrate the benefit of early initiation of ART, it demonstrated feasibility of initiating ART in PWH who are receiving care in the ICU. More research is needed to determine the impact of rapid start ART in PWH who are critically ill.

## CONTINUATION OF ART IN PWH WHO ARE CRITICALLY ILL

Up to 70% of PWH are already receiving ART upon ICU admission [10]. However, deciding whether to continue ART in critically ill patients represents a clinical conundrum with limited guidance [1, 13]. Determining whether the ART represents a complete regimen often requires discussing medical history with patients, patients' care partners, clinicians, or pharmacists, and reviewing outpatient or prior outpatient and hospital records.

Moreover, critically ill patients often experience acute renal or hepatic dysfunction, which may require antiretroviral medication dosage adjustments. The absorption, distribution, metabolism, and elimination of antiretroviral medications can be altered in these patients, posing a challenge due to limited data on the complex interplay between ART, patient factors, and treatment approaches used to manage critical illnesses. Additional factors that may influence the continuation of ART include adverse drug reactions, drug interactions, lack of enteral access, ability to administer via enteral feeding tube (ability to crush or availability in a liquid formulation), and impaired enteral absorption. Careful consideration and evaluation should be given to PWH who are coinfected with hepatitis B virus (HBV), as discontinuation of ART can lead to HBV rebound or “flare” [28, 29]. Furthermore, many healthcare institutions have a limited selection of ART options on their inpatient formulary, which may require therapeutic interchange based on formulary guidelines or splitting components of STRs. Importantly, within-class interchanges may be problematic due to differences in barriers to resistance, drug interactions, and adverse events. For some patients, the best option may be to have their ART used in the outpatient setting brought in to be administered while hospitalized, although this approach presents logistical barriers and potential risks for errors.

There have been multiple studies that assessed timing of ART initiation in patients who are newly diagnosed and in relation to specific OIs [30–33]. However, there is little guidance on the continuation of ART during critical illness. Expert opinion recommends continuing ART throughout critical illness if ART can be safely administered with minimal risk of drug toxicity or drug interactions [16, 34, 35]. Additionally, the US Department of Health and Human Services (HHS) guideline for management of HIV suggests that stopping ART for a short time (eg, <1 to 2 days) usually can be done by holding all drugs in the regimen [21]. Reasons for short-term interruption include illnesses that preclude oral intake, surgical procedures, drug toxicity, or interrupted access to ART. Furthermore, if a patient experiences a severe or life-threatening toxicity to ART, all components of the regimen should be stopped simultaneously. The timing of reinitiating a different regimen that does not include the offending agent(s) after toxicity resolves should be evaluated on a case-by-case basis. However, the HHS recommends that all efforts should be made to minimize duration of ART discontinuation. This is underscored by a recent in-depth analysis where delaying a single dose of long-acting cabotegravir/rilpivirine resulted in virologic failure [36].

Data evaluating continuation of ART in the ICU are limited and conflicting. Several studies have not found a significant difference in outcomes between patients who were continued on ART and those who were not during ICU admission [37–40]. Alternatively, some have suggested a benefit of continuing ART during critical illness. A retrospective study conducted in France from January 2000 to December 2009 included 85 PWH across 91 admissions, primarily for respiratory failure and neurological disorders, and found no difference in mortality at the time of ICU discharge between those who continued ART and those who did not (16% vs 20%, *P* = .78) [41]. Unadjusted survival over 6 months was higher in patients who continued ART (*P* = .04) with a decreased incidence of new AIDS-related events at 6 months (17% vs 34%, *P* = .07). Rates of resistance to ART at 6 months were significantly higher among those who continued ART in the ICU (25% vs 7%, *P* = .02), which was thought to be due to a decrease in gastrointestinal absorption of ART during critical illness or lack of adherence after discharge from the ICU. While the increased prevalence of resistance is concerning, only 52% of patients were receiving ART at ICU admission and virologic data for each of these cases were not provided. In a separate study from France from 1997 to 2008, continuation or initiation of ART was associated with improved outcomes based on a retrospective study of 98 PWH who required ICU admission [42]. Fewer PWH with no history of ART survived ICU admission compared to those who started or continued ART (33% vs 20%). Introduction or continuation of ART was significantly associated with improved ICU-related outcomes after multivariate analysis (odds ratio [OR], 0.28 [95% confidence interval {CI}, .08–.94]; *P* = .04).

A meta-analysis performed through 2017 including 12 single-center retrospective studies comprising 1584 PWH was conducted to evaluate the impact of ART continuation on ICU-related mortality [43]. Reduction in short-term mortality was observed following initiation or continuation of ART in the ICU (OR, 0.53 [95% CI, .31–.91]; *P* = .02). Of the included studies, only 6 reported long-term (≥90 days) follow-up. Improved outcomes were reported in 3 studies at 6 [44], 12 [42], and 60 months [45], whereas the other 3 failed to demonstrate any long-term benefit.

More recently, a retrospective analysis of 110 PWH admitted to the ICU in Colombia from 2017 to 2019 reported a decreased mortality in those on ART prior to admission [46]. Logistic regression analysis found that absence of ART on ICU admission was associated with a significantly higher odds of mortality (OR, 2.5 [95% CI, 1.0–6.1]; *P* = .037). Indeed, ART was determined to be the main determinant of mortality regardless of ICU length of stay based on findings from a predictive model of mortality.

Though data supporting improvements in long-term mortality among PWH who require ICU admission are lacking, long-term benefits of ART in nonhospitalized PWH are irrefutable. In addition, the most common causes of ICU admission for PWH are vastly different due to improvements in modern ART [1] compared to the study periods discussed above. If ART is inadvertently or purposefully interrupted during ICU admission, it is crucial to discontinue all components of ART as well as understand possible adverse events and potential consequences associated with interruptions, such as acute worsening of viral coinfections, development of resistance, and progression of HIV leading to immunodeficiencies and OIs.

Admission to the ICU can disrupt the dosing schedules of LAI formulations of cabotegravir, rilpivirine, and lenacapavir. Maintenance doses of cabotegravir and rilpivirine should be administered within 7 days of the planned monthly date [47, 48]. If the interruption exceeds 7 days, oral cabotegravir and rilpivirine can be substituted for the missed injections for up to 2 consecutive months. However, for patients requiring oral therapy for longer than 2 months, an alternative regimen, potentially including rilpivirine, is recommended. For both monthly and bimonthly dosing regimens, if the missed dose occurred within the past month (28 days), the original dosing schedule can be resumed. If the lapse in treatment extends beyond 1 month (28 days), then therapy should be restarted with the initiation dose followed by continuation doses. For patients experiencing subcutaneous lenacapavir injection interruptions, weekly oral lenacapavir 300 mg can serve as a bridging strategy until resumption of subcutaneous injections [49].

Much of the available evidence is not indicative of complications associated with short-term interruptions of therapy. However, the benefits of continuing ART during ICU admission generally outweigh the risk, and if ART is stopped, it should be reinitiated as soon as possible.

## ALTERNATIVE ROUTES OF ANTIRETROVIRAL ADMINISTRATION

PWH most often require ICU admission due to acute respiratory failure, of which approximately 50% require intubation with mechanical ventilation [50]. Enteral feeding tubes are often placed when intubation is completed or in individuals with the inability to swallow, allowing for medications to be administered via tube by crushing 1 or more tablets or utilizing a liquid formulation, depending on the tube diameter. Liquid formulations are available for many older antiretrovirals (eg, lamivudine [3TC], lopinavir/ritonavir) but are typically formulated for pediatric use and require large volumes for use in adults, potentially leading to increased exposure to excipients (eg, abacavir [ABC] and nevirapine suspensions contain 340 mg and 162 mg of sorbitol/mL, respectively; lopinavir/ritonavir solution contains 2.12 mL of alcohol and 763 mg of propylene glycol/5 mL [51]) and higher rates of adverse events. Routinely flushing enteral feeding tubes, especially before and after ART administration, will decrease the proportion of antiretroviral remaining in the tube and may prevent tube blockage. To mimic fasting conditions and ensure optimal absorption for certain ART regimens, enteral feedings should be held for 30 minutes before and 1 hour after ART administration, especially with INSTIs due to the risk of chelation with polyvalent cations [51, 52]. However, some ART regimens may combine antiretrovirals with different absorption requirements, some needing fasting and others needing food, potentially requiring dose separation.

In the past, many manufacturers of antiretroviral medications did not provide recommendations for crushing, splitting, or opening oral dosage forms, raising concerns as to whether modification of oral dosage forms may alter pharmacokinetics, leading to impaired virologic response. Few modern antiretroviral medications have been studied for bioequivalence when crushed or when administered as each individual component, and the available studies were performed in healthy people without HIV (Table 1) [53, 57, 65, 68, 69]. Recently, an evaluation was performed in people without HIV to determine bioequivalence of bictegravir (BIC)/emtricitabine (FTC)/tenofovir alafenamide (TAF) when administered whole, crushed, or dissolved [54, 55, 58–60, 63, 64, 66, 67, 70]. The area under the curve and maximum concentration of TAF and FTC were lower when tablets were crushed, but bioequivalence was demonstrated when tablets were dissolved in water prior to administration.

**Table 1.** Alternative Routes of Administration and Dose Adjustments for Modern Antiretroviral Therapies

Generic Components (Brand)	Can Tablets Be Crushed?	Comments on Crushing or Dissolving Tablets	Renal Dose Adjustments [21]	Hepatic Dose Adjustments [21]
BIC/FTC/TAF (Biktarvy)	No, tablets should be dissolved in water prior to administration as crushed tablets may result in suboptimal FTC/TAF exposures.	A phase 1 study in healthy people without HIV examined the dissolution of a tablet in water or crushed in applesauce compared to a whole tablet [53]. Dissolving the tablet achieved bioequivalence, whereas crushing the tablet resulted in lower FTC and TAF exposure. While some reports indicate that crushed BIC/FTC/TAF maintained virologic suppression [54, 55], including in critically ill PWH unable to swallow oral ART [56], others reported virologic failure and development of resistance in PWH with uncontrolled viremia who crushed BIC/FTC/TAF prior to administration [54, 55].	Not recommended in CrCl <30 mL/min unless receiving IHD.	Not recommended in Child-Pugh class C hepatic dysfunction.
DTG/ABC/3TC (Triumeq)	Yes	A bioequivalence study in healthy people without HIV demonstrated higher DTG plasma concentrations when DTG/ABC/3TC tablets were crushed and suspended before administration, which was not considered clinically relevant [57]. Previous case reports describe adequate concentrations when DTG/ABC/3TC tablets were crushed and administered via nasogastric tube, resulting in virologic suppression [58, 59]. DTG tablets can be crushed, but enteral feedings should be held for 2 h before and after administration [60, 61]; 5-mg dispersible tablets are also available. DTG dose increases may be required to achieve adequate exposure [60]. Liquid formulations of ABC and 3TC are available.	Not recommended in CrCl <30 mL/min; limited data supporting use in IHD [62].	Contraindicated in Child-Pugh class B or C hepatic dysfunction due to ABC component.
DTG/3TC (Dovato)	No	The manufacturer recommends against crushing. DTG tablets can be crushed but enteral feedings should be held for 2 h before and after administration [60, 61]; 5-mg dispersible tablets are also available. DTG dose increases may be required to achieve adequate exposure [60]. A liquid formulation of 3TC is available.	Not recommended in CrCl <50 mL/min; may use clinical judgment to extrapolate data from DTG/ABC/3TC for use in IHD.	Not recommended in Child-Pugh class C hepatic dysfunction.
EVG/COBI/FTC/TAF (Genvoya)	No. Tablets should be dissolved prior to administration as crushed tablets may result in suboptimal FTC/TAF exposures.	A bioequivalence study in healthy people without HIV identified lower, but clinically insignificant, concentrations of EVG when EVG/COBI/FTC/TAF was crushed and administered with breakfast or enteral nutrition compared to administration as a whole tablet [53]. A case report described a PWH who was newly diagnosed and able to achieve and maintain virologic suppression despite crushing EVG/COBI/FTC/TAF tablets due to difficulty swallowing whole tablets [63]. Alternatively, a bioequivalence study in healthy people without HIV demonstrated that dissolving EVG/COBI/FTC/TAF tablets achieved comparable FTC concentrations compared to whole tablet [64]. However, concentrations of TAF and FTC were lower when EVG/COBI/FTC/TAF tablets were dissolved, though the clinical significance is unknown.	Not recommended in CrCl <30 mL/min unless receiving IHD.	Not recommended for patients with severe hepatic impairment.
DRV/COBI/FTC/TAF (Symtuza)	No	A bioequivalence study in healthy people without HIV demonstrated comparable AUC and C_max_ when DRV/COBI/FTC/TAF tablets were split or administered whole [65]. However, concentrations of TAF and FTC were lower when DRV/COBI/FTC/TAF tablets were crushed prior to administration, though the clinical significance is unknown.	Not recommended in CrCl <30 mL/min; may use clinical judgment to extrapolate data from other FTC/TAF-containing single-tablet regimens for use in IHD.	Not recommended for patients with severe hepatic impairment.
DOR/TDF/3TC (Delstrigo)	No	A case report described a PWH who maintained virologic suppression on ART containing DOR that was crushed and mixed with 60 mL of water prior to administration via nasojejunal tube [66]. TDF tablets can be crushed and dissolved in water [61]; an oral powder formulation is also available. A liquid formulation of 3TC is available.	Not recommended in CrCl <50 mL/min.	None
RPV/FTC/TAF (Odefsey)	No	Not studied. RPV is insoluble in water and may lead to decreased trough concentrations when crushed and administered with water [60], by adding to semisolid foods, administering with enteral feedings, or increasing the RPV dose [60, 67].	Not recommended in CrCl <30 mL/min unless receiving IHD—administer after IHD on dialysis days.	None
DTG/RPV (Juluca)	No	DTG tablets can be crushed but enteral feedings should be held for 2 h before and after administration [60, 61]; 5-mg dispersible tablets are also available. DTG dose increases may be required to achieve adequate exposure [60]. RPV is insoluble in water and may lead to decreased trough concentrations when crushed and administered with water [60], which may be overcome by adding to semisolid foods, administering with enteral feedings, or increasing the RPV dose [60, 67].	None	None
CAB/RPV long-acting injectable (Cabenuva)	NA	Administered as an intramuscular injection.	None	None

For further details, refer to the following online resources: “Oral Antiretroviral/HCV DAA Administration: Information on Crushing and Liquid Drug Formulations” (https://www.hivclinic.ca/main/drugs_extra_files/Crushing%20and%20Liquid%20ARV%20Formulations.pdf), the University of Liverpool's “ARV formulations for Swallowing Difficulties” (https://liverpool-hiv-hep.s3.amazonaws.com/prescribing_resources/pdfs/000/000/011/original/ARV_Swallowing_2018_Oct.pdf), and the European AIDS Clinical Society’s “ARVs: Swallowing Difficulties” (https://eacs.sanfordguide.com/drug-drug-interactions-other-prescribing-issues/other-prescribing-issues/eacs-arvs-persons-with-swallowing-difficulties).

Abbreviations: 3TC, lamivudine; ABC, abacavir; ART, antiretroviral therapy; AUC, area under the curve; BIC, bictegravir; CAB, cabotegravir; COBI, cobicistat; C_max_, maximum concentration; CrCl, creatinine clearance; DOR, doravirine; DRV, darunavir; DTG, dolutegravir; EVG, elvitegravir; FTC, emtricitabine; HIV, human immunodeficiency virus; IHD, intermittent hemodialysis; NA, not applicable; PWH, people with human immunodeficiency virus; RPV, rilpivirine; TAF, tenofovir alafenamide; TDF, tenofovir disoproxil fumarate.

Most real-world efficacy and safety data for modifying oral formulations of various ART are based on case reports and series that demonstrate achieving or maintaining virologic responses [53]. However, in a recent retrospective study of 53 critically ill PWH (96% treatment experienced, 78% virologically suppressed) unable to swallow oral ART, crushed ART with either dolutegravir (DTG) as part of ART (DTG = 70%) or BIC as part of BIC/FTC/TAF (BIC = 30%) maintained virologic suppression in most patients [56]. Mechanical ventilation (83%) was the primary indication for crushed ART. The median duration for crushed ART was 10.5 days (first quartile–third quartile, 3.25–18.75 days) with 43% of patients receiving all doses without interruption. Missed doses, more frequent with DTG regimens (66.7% vs 33.3% for BIC), resulted in a median of 2 missed doses per patient overall, primarily due to nothing by mouth orders or feeding tube issues. These findings provide real-world support for the clinical utility of crushed DTG- or BIC-containing regimens for virologically suppressed PWH in critical care settings.

However, reports of some PWH with uncontrolled viremia who crushed BIC/FTC/TAF tablets for administration experienced virologic failure and developed treatment-emergent resistance [54, 55, 71]. While others reported maintenance of virologic suppression [56, 64], BIC/FTC/TAF tablets should be dissolved prior to administration as crushed tablets may result in suboptimal FTC/TAF exposures [53].

Modifying oral formulations of ART is viable option for administration in critically ill PWH who are unable to use the oral route. It is important to recognize that preparation of ART for administration via enteral tube should be individualized based on the specific components as data are unavailable for some oral formulations of antiretrovirals, whereas others cannot be modified. Ideally, these adjustments should be overseen by infectious diseases or HIV specialists or by clinical pharmacists with expertise in ART. Several online resources provide valuable guidance, including “Oral Antiretroviral/HCV DAA Administration: Information on Crushing and Liquid Drug Formulations” (https://www.hivclinic.ca/main/drugs_extra_files/Crushing%20and%20Liquid%20ARV%20Formulations.pdf), the University of Liverpool's “ARV Formulations for Swallowing Difficulties” (https://liverpool-hiv-hep.s3.amazonaws.com/prescribing_resources/pdfs/000/000/011/original/ARV_Swallowing_2018_Oct.pdf), and the European AIDS Clinical Society’s “ARVs: Swallowing Difficulties” (https://eacs.sanfordguide.com/drug-drug-interactions-other-prescribing-issues/other-prescribing-issues/eacs-arvs-persons-with-swallowing-difficulties). These resources offer information on crushing tablets and formulating liquid ART medications. Since many of these resources are in PDF format, it is recommended to bookmark these links, as they are frequently updated with new information. In regions where such expertise is scarce, the National Clinician Consultation Center, at the University of California, San Francisco, provides free teleconsultation services, offering expert guidance on preventing and treating HIV, from treatment initiation to advanced disease management.

## ANTIRETROVIRAL INTERACTIONS WITH DISEASE STATES IN PWH WHO ARE CRITICALLY ILL

Critically ill PWH in the ICU with multiorgan failure and hemodynamic instability may experience changes in hepatic and renal function, which may lead to drug–disease interactions requiring dose adjustments of antiretrovirals.

While hepatotoxicity remains a potential concern with ART, the risk associated with modern regimens is significantly lower compared to older, less frequently used drugs [72]. However, the presence of hepatic insufficiency poses additional considerations. While most antiretrovirals do not require dosage adjustments in mild liver impairment (Child-Pugh class A), exceptions exist for specific drugs (Table 1) [21].

Acute kidney injury (AKI) has been associated with increased morbidity and mortality in PWH, and identification of modifiable risk factors for AKI has remained difficult [73]. Renal replacement therapy (RRT) in the ICU is most commonly provided as continuous renal replacement therapy (CRRT) or intermittent hemodialysis [74]. The choice of RRT is typically guided by hemodynamic status, amount of fluid removal required, mobility of patient, and electrolyte control, among others. Modern ART consists of STRs or LAIs containing multiple antiretrovirals, which may require dose adjustments of individual antiretrovirals in the setting of AKI and/or RRT [21]. To optimize ART and minimize risk, identifying the specific RRT modality used is crucial for determining necessary adjustments.

Nucleoside reverse transcriptase inhibitors (NRTIs) are the primary antiretroviral class that require renal dose adjustments and are components of a majority of STRs [21]. Currently, 3 STRs are approved by the US Food and Drug Administration in PWH undergoing hemodialysis: BIC/FTC/TAF, elvitegravir/cobicistat/FTC/TAF, and darunavir/cobicistat/FTC/TAF [75–77]. Additionally, the STR DTG/ABC/3TC has demonstrated safety and efficacy in a small cohort of PWH receiving intermittent hemodialysis for end-stage renal disease [62]. Extrapolation of these data on safety and efficacy of FTC, 3TC, and TAF in PWH on RRT to all formulations containing these NRTIs is practiced by some HIV clinicians and has been studied in a small cohort of PWH on FTC/TAF-containing STRs [78]. The primary NRTI requiring renal dose adjustment is tenofovir disoproxil fumarate (TDF) in PWH with creatinine clearance (CrCl) ≤50 mL/minute or in those with CrCl ≤70 mL/minute when used in combination with cobicistat due to the higher risk of renal toxicity from TDF in boosted regimens [79–81]. Package inserts for some antiretroviral medications, such as 3TC, recommend dose adjustments for PWH who have renal dysfunction, but standard doses are well tolerated and commonly used to promote adherence [82].

On the other hand, continuous venovenous hemofiltration (CVVH) is a commonly used modality for CRRT in the ICU and may lead to increased clearance of renally eliminated antiretrovirals [83]. In addition, augmented renal clearance, defined as an estimated glomerular filtration rate ≥130 mL/minute/1.73 m^2^, is a commonly occurring phenomenon in the ICU and may also impact clearance of renally eliminated medications [84]. While other classes of antiretroviral medications are primarily eliminated hepatically or extrahepatically, NRTIs remain of concern as they are eliminated renally, except for ABC, which undergoes hepatic metabolism via alcohol dehydrogenase and glucuronyl transferase [21]. Increased clearance of NRTIs may lead to decreased drug concentrations, which impact ART efficacy and increase risk for drug resistance [21, 83]. Proposed guidelines on dosing of NRTIs based on CVVH flow rate (L/hour) have been published, but later pharmacokinetic studies demonstrated inaccuracies in this approach, with only 41% of NRTIs providing a favorable pharmacokinetic profile [83, 85]. Further studies are needed to demonstrate safety and efficacy of renally eliminated antiretroviral medications in the setting of increased renal clearance.

## INTERACTIONS BETWEEN ANTIRETROVIRALS AND COMMONLY USED DRUGS FOR PWH WHO ARE CRITICALLY ILL IN THE ICU

Throughout an ICU admission, clinical management changes acutely and commonly requires balancing of multiple comorbidities. For PWH receiving ART prior to ICU admission, various drug interactions exist that may clinically alter the efficacy of ART or increase the risk of adverse events from increased concentrations of antiretrovirals or other medications commonly used in the ICU (Table 2) [1, 21, 51, 86–89]. Fortunately, the safety profile and reduced interaction potential of modern ART have improved significantly. While older antiretrovirals like NNRTIs, protease inhibitors, and pharmacoenhancers (eg, ritonavir or cobicistat) are less commonly used, notable interactions remain, particularly with INSTIs and LAIs. Commonly administered antacids and supplements in the ICU, often considered benign, can chelate with INSTIs, reducing their absorption and efficacy [51]. Separation of INSTIs and polyvalent cations is often required to avoid this interaction. Additionally, the extended half-life of LAIs, like lenacapavir, can prolong potential drug interactions for months after the last dose [88, 89]. These interactions may go unnoticed unless meticulously documented in the electronic medical record or disclosed during medication reconciliation.

**Table 2.** Interactions Between Antiretrovirals and Commonly Used Drugs for People With Human Immunodeficiency Virus Who Are Critically Ill in the Intensive Care Unit

Medications Commonly Used in the ICU	ARV class	ARV medication	Outcome of Interaction	Comments
Polyvalent cations Multivitamins Iron supplements Phosphate binders (eg, calcium acetate) Antacids (eg, sucralfate, aluminum hydroxide, calcium carbonate)	INSTI	Bictegravir	↓ INSTI	Polyvalent cations are common due to enteral feeding, electrolyte management, and other reasons. Each INSTI and polyvalent cation medication should be individually assessed. Based on the INSTI and type of polyvalent cation, timing of separation or coadministration may differ significantly. Aluminum and calcium-free phosphate binders (eg, sevelamer, lanthanum) may be feasible, especially if separate administration times are not possible.
		Cabotegravir (PO)		
		Dolutegravir		
		Elvitegravir		
		Raltegravir		
Anticonvulsants Carbamazepine Oxcarbazepine Fos/phenytoin Phenobarbital	INSTI	Bictegravir	↓ INSTI	Concomitant use of carbamazepine, oxcarbazepine, fos/phenytoin, and phenobarbital is not recommended. Dolutegravir may be increased to 50 mg twice daily in ART naive. Do not administer in those with INSTI experience and have known or suspected INSTI resistance. Anticonvulsant selection should be carefully considered in those on ART, especially since both may be chronic therapies and anticonvulsants may have prolonged half-lives.
		Cabotegravir (PO, IM)		
		Dolutegravir		
		Elvitegravir/cobicistat		
		Raltegravir		
	NNRTI	Rilpivirine (PO, IM)	↓ rilpivirine	Concomitant use of carbamazepine, oxcarbazepine, fos/phenytoin, and phenobarbital is not recommended. Anticonvulsant selection should be carefully considered in those on ART, especially since both may be chronic therapies and anticonvulsants may have prolonged half-lives.
	NRTI	Tenofovir alafenamide	↓ tenofovir alafenamide	Concomitant use of carbamazepine, oxcarbazepine, fos/phenytoin, and phenobarbital is not recommended. Anticonvulsant selection should be carefully considered in those on ART, especially since both may be chronic therapies and anticonvulsants may have prolonged half-lives.
	PI	Darunavir/cobicistat	↓ darunavir/cobicistat	Concomitant use of carbamazepine, oxcarbazepine, fos/phenytoin, and phenobarbital is not recommended. Anticonvulsant selection should be carefully considered in those on ART, especially since both may be chronic therapies and anticonvulsants may have prolonged half-lives.
		Darunavir/ritonavir	↓ darunavir/ritonavir	Concomitant use of oxcarbazepine, fos/phenytoin, and phenobarbital is not recommended. Anticonvulsant selection should be carefully considered in those on ART, especially since both may be chronic therapies and anticonvulsants may have prolonged half-lives.
			↑ carbamazepine	Monitor carbamazepine concentrations and adjust dose if needed. Darunavir/ritonavir may be increased to twice daily.
	Entry inhibitor	Fostemsavir	↓ temsavir (the active metabolite of fostemsavir)	Concomitant use of carbamazepine, oxcarbazepine, fos/phenytoin, and phenobarbital is not recommended. Anticonvulsant selection should be carefully considered in those on ART, especially since both may be chronic therapies and anticonvulsants may have prolonged half-lives.
	Capsid inhibitor	Lenacapavir	↓ lenacapavir	Concomitant use of carbamazepine, oxcarbazepine, fos/phenytoin, and phenobarbital is not recommended. Anticonvulsant selection should be carefully considered in those on ART, especially since both may be chronic therapies and anticonvulsants may have prolonged half-lives. The extended half-life of lenacapavir can prolong potential drug interactions for months.
Antiplatelets Clopidogrel Ticagrelor Vorapaxar	INSTI	Elvitegravir/cobicistat	↓ clopidogrel active metabolite	Potent CYP inhibitors (cobicistat) will alter conversion of clopidogrel (prodrug) into active metabolite. Consider other antiplatelets (eg, prasugrel).
			↑ ticagrelor	Administration of ticagrelor with CYP inhibitors is not recommended and may increase ticagrelor exposure. Consider other antiplatelets (eg, prasugrel).
			↑ vorapaxar	Administration of vorapaxar with CYP inhibitors is not recommended and may increase vorapaxar exposure. Consider other antiplatelets (eg, prasugrel).
	PI	Darunavir/ritonavir Darunavir/cobicistat	↓ clopidogrel active metabolite	Potent CYP inhibitors (cobicistat) will alter conversion of clopidogrel (prodrug) into active metabolite. Consider other antiplatelets (eg, prasugrel).
			↑ ticagrelor	Administration of ticagrelor with CYP inhibitors is not recommended and may increase ticagrelor exposure. Consider other antiplatelets (eg, prasugrel).
			↑ vorapaxar	Administration of vorapaxar with CYP inhibitors is not recommended and may increase vorapaxar exposure. Consider other antiplatelets (eg, prasugrel).
	Capsid inhibitor	Lenacapavir	↓ clopidogrel active metabolite	Moderate CYP3A4 inhibitors (lenacapavir) may alter conversion of clopidogrel (prodrug) into active metabolite. Consider other antiplatelets (eg, prasugrel) The extended half-life of lenacapavir can prolong potential drug interactions for months.
			↑ ticagrelor	Administration of ticagrelor with CYP3A4 inhibitors is not recommended and may increase ticagrelor exposure. Consider other antiplatelets (eg, prasugrel) The extended half-life of lenacapavir can prolong potential drug interactions for months.
Cardiovascular Amiodarone Dofetilide Ranolazine Ivabradine	INSTI	Bictegravir	↑ amiodarone, dofetilide, ranolazine, and ivabradine	Concomitant use of bictegravir or dolutegravir and dofetilide is not recommended. Concomitant use of elvitegravir/cobicistat and these cardiovascular agents is not recommended.
		Dolutegravir		
		Elvitegravir/cobicistat		
	PI	Darunavir/ritonavir Darunavir/cobicistat	↑ amiodarone, dofetilide, ranolazine, and ivabradine	Concomitant use of darunavir-containing ART and these cardiovascular agents is not recommended unless benefits outweigh risks. Monitor closely if utilized.
	Entry inhibitor	Fostemsavir	↑ temsavir (the active metabolite of fostemsavir)	Monitor for QTc interval prolongation and ventricular arrythmias when lenacapavir is used with amiodarone or dofetilide.
	Capsid inhibitor	Lenacapavir	↑ amiodarone, ranolazine, and ivabradine	Concomitant use of lenacapavir and amiodarone or ivabradine is not recommended. Careful dose titration of ranolazine is recommended. The extended half-life of lenacapavir can prolong potential drug interactions for months.
Narcotics and sedatives Fentanyl IV Midazolam IV	INSTI	Elvitegravir/cobicistat	↑ narcotic and sedative effects	Consider careful titration and use validated monitoring scales for pain/sedation.
	PI	Darunavir/ritonavir Darunavir/cobicistat	↑ narcotic and sedative effects	Consider careful titration and use validated monitoring scales for pain/sedation.
	Capsid inhibitor	Lenacapavir	↑ narcotic and sedative effects	Consider careful titration and use validated monitoring scales for pain/sedation.
Pulmonary hypertension Sildenafil Tadalafil	INSTI	Elvitegravir/cobicistat	↑ PDE 5 inhibition	Concomitant use of sildenafil is not recommended. Tadalafil may be an alternative at lower dose and should be titrated.
	PI	Darunavir/ritonavir Darunavir/cobicistat	↑ PDE 5 inhibition	Concomitant use of sildenafil is not recommended. Tadalafil may be an alternative at lower dose and should be titrated.
	Capsid inhibitor	Lenacapavir	↑ PDE 5 inhibition	Sildenafil and tadalafil should be started at lower doses and then titrated.
Acid-suppressive therapies Antacids H_2_-receptor antagonists Proton pump inhibitors	NNRTI	Rilpivirine (PO)	↓ rilpivirine	Acid-suppressive therapies commonly used in critically ill patients for various reasons, including stress ulcer prophylaxis. Each NNRTI and acid-suppressive therapy should be individually assessed. Antacids may be separated whereas proton pump inhibitors are considered a contraindication. Rilpivirine should be administered with meals to increase absorption.
Anticoagulants Apixaban Rivaroxaban	INSTI	Elvitegravir/cobicistat	↑ anticoagulant	Dose-reduced apixaban may be used on a case-by-case basis. Avoid in those requiring apixaban 2.5 mg twice daily. In those on apixaban 5 to 10 mg twice daily, consider reducing dose by 50%. Avoid in those requiring rivaroxaban.
	PI	Darunavir/cobicistat Darunavir/ritonavir	↑ anticoagulant	Dose-reduced apixaban may be used on a case-by-case basis. Avoid in those requiring apixaban 2.5 mg twice daily. In those on apixaban 5 to 10 mg twice daily, consider reducing dose by 50%. Avoid in those requiring rivaroxaban.
	Capsid inhibitor	Lenacapavir	↑ anticoagulant	Monitor for evidence of bleeding. Rivaroxaban does not require dose adjustment if CrCl >80 mL/min, but is not recommended if CrCl 15–80 mL/min. The extended half-life of lenacapavir can prolong potential drug interactions for months.
Agitation/delirium Haloperidol Olanzapine Quetiapine	NNRTI	Rilpivirine (PO)	↔ haloperidol	Monitor for QTc interval prolongation.
	PI	Darunavir/cobicistat Darunavir/ritonavir	↑ haloperidol, quetiapine	Monitor for increased haloperidol or quetiapine concentrations, as well as associated toxicities, including QTc prolongation. If coadministration is necessary, adjust the quetiapine dosage to one-sixth of the standard dose.
	INSTI	Elvitegravir/cobicistat	↑ haloperidol, quetiapine	Monitor for increased haloperidol or quetiapine concentrations, as well as associated toxicities, including QTc prolongation. If coadministration is necessary, adjust the quetiapine dosage to one-sixth of the standard dose.
	Capsid inhibitor	Lenacapavir	↑ quetiapine	Monitor for increased quetiapine concentrations, as well as associated toxicities, including QTc prolongation. If coadministration is necessary, consider reducing the dosage of quetiapine.
Glucocorticoids Betamethasone (local injections) Betamethasone (systemic) Budesonide (inhaled or intranasal) Budesonide (systemic) Ciclesonide (inhaled or intranasal) Dexamethasone (systemic) Fluticasone (inhaled or intranasal) Methylprednisolone (local injections) Mometasone (inhaled or intranasal) Prednisone (systemic) Triamcinolone (local injections)	NNRTI	Rilpivirine (PO)	↓ rilpivirine	Concomitant use of rilpivirine-containing ART with multiple doses of dexamethasone is not recommended.
	PI	All PIs	↑ glucocorticoids ↓ PI	Concomitant use of PI-containing ART with inhaled or intranasal budesonide, ciclesonide, fluticasone, and mometasone, as well as systemic betamethasone, budesonide, or locally administered betamethasone, methylprednisolone, and triamcinolone injections is not recommended. If coadministration is necessary, consider an alternative or dose-reduced glucocorticoid.
				Concomitant use of PI-containing ART with systemic betamethasone, budesonide, or dexamethasone may decrease concentrations of PIs. If coadministration is necessary, consider an alternative glucocorticoid for long-term use and monitor the virologic response to ART.
	INSTI	Elvitegravir/cobicistat	↑ glucocorticoids	Concomitant use of INSTI-containing ART with inhaled or intranasal budesonide, ciclesonide, fluticasone, and mometasone, as well as systemic budesonide, or locally administered betamethasone and triamcinolone injections is not recommended. If coadministration is necessary, consider an alternative or dose-reduced glucocorticoid. Consider an alternative or reduced dose if coadministration of systemic betamethasone or locally administered methylprednisolone injections is necessary.
		Bictegravir Elvitegravir/cobicistat	↓ bictegravir ↓ elvitegravir	Concomitant use of bictegravir- or elvitegravir/cobicistat-containing ART with systemic betamethasone, budesonide, or dexamethasone may decrease concentrations of bictegravir and elvitegravir. If coadministration is necessary, consider an alternative glucocorticoid for long-term use and monitor the virologic response to ART.
	Capsid inhibitor	Lenacapavir	↑ glucocorticoids ↓ lenacapavir	If coadministration of lenacapavir-containing ART with inhaled or intranasal budesonide, fluticasone, mometasone, as well as systemic budesonide, prednisone, or dexamethasone, or locally administered betamethasone, methylprednisolone, or triamcinolone injections is necessary, consider an alternative or dose-reduced glucocorticoid.
				Concomitant administration of systemic dexamethasone at doses equal to or exceeding 16 mg/d may lead to reduced concentrations of lenacapavir and is not recommended.

Sources: [1, 21, 51, 86–89].

Abbreviations: ART, antiretroviral therapy; CrCl, creatinine clearance; CYP, cytochrome P450; ICU, intensive care unit; IM, intramuscular; INSTI, integrase strand transfer inhibitor; IV, intravenous; NNRTI, nonnucleoside reverse transcriptase inhibitor; NRTI, nucleoside reverse transcriptase inhibitor; PDE, phosphodiesterase; PI, protease inhibitor; PO, by mouth.

ARVs and glucocorticoids can have significant drug interactions. While most research has focused on inhaled glucocorticoids and pharmacoenhancers [90, 91], the use of systemic glucocorticoids with concurrent pharmacoenhancers requires caution [92]. The potential benefits must be weighed against the risks of a Cushingoid response, particularly with short-term intravenous therapy.

Dexamethasone presents a unique challenge due to its dual role as a substrate and a dose-dependent inducer of the CYP3A4 enzyme [92]. This dual effect creates a variable risk of drug interactions depending on the dexamethasone dosage regimen. Dexamethasone may decrease concentrations of all NNRTIs and compromise virologic efficacy, especially rilpivirine. Due to this increased risk, more than a single dose of dexamethasone is contraindicated for patients taking rilpivirine. Systemic dexamethasone may also decrease concentrations of bictegravir, cobicistat-boosted elvitegravir, and lenacapavir. Further, lenacapavir concentrations may decrease when dexamethasone doses exceed 16 mg per day. Dose adjustments should be continued for a minimum of 2 weeks after discontinuation of dexamethasone treatment, accounting for its persistent inducing effect postdiscontinuation.

Recognition of potential interactions with antiretrovirals and commonly used drugs for PWH who are critically ill in the ICU is critical to avoid patient harm. A recent single-center retrospective study identified 208 drug interactions from 53 of 77 adult PWH on ART who required ICU care for at least 24 hours [93]. The mean number of interactions was 4 ± 2 per ICU admission, and antipsychotics and analgesics were most often implicated. Of these interactions, 12% were contraindicated and 65% were considered major. Utilization of drug interaction resources, including HHS guidelines and the University of Liverpool's ART web-based resource [21, 51], is recommended for PWH on ART who require ICU admission.

## ANTIRETROVIRAL STEWARDSHIP IN THE ICU

Despite the diverse etiologies of ICU admissions among PWH, guidance on pharmacotherapy-related decision-making tools specifically tailored to this critically ill patient population are lacking. A recent article emphasized the need for ART stewardship and provided recommendations for program consideration and implementation, highlighting the gap in literature compared to other commonly used medications for infectious diseases [94]. The proposed core elements for antiretroviral stewardship programs (ARVSPs) emphasized leadership commitment, accountability, drug expertise, key support, tracking, reporting, and comprehensive education. Adequate full-time equivalent allocation and a dedicated project leader were deemed essential for successful ARVSP implementation. Expertise in HIV pharmacotherapy was also considered crucial. Key support systems included information technology, formulary management, and socioeconomic considerations to ensure accessibility after discharge. Tracking and reporting of medication-related errors, pharmacist intervention, and related outcomes were also recommended. As with any impactful protocol, education remained a cornerstone of ARVSP success, encompassing the patient, provider, pharmacist, nurse, and social worker.

The implementation of ARVSPs has yielded significant benefits by minimizing medication errors and promptly resolving them before discharge [95–97]. A single-center study of approximately 120 PWH demonstrated the effectiveness of a pharmacist-led ARVSP in reducing medication error rates associated with ART and OI medications, as well as significantly lowering 30-day all-cause readmission rates and increasing linkage to care rates [98]. These findings highlight the potential of ARVSPs in improving patient outcomes, but further research is needed to develop more granular considerations and decision tools for ART administration, drug interactions, and disease state interactions.

## CONCLUSIONS

Ensuring optimal patient outcomes and safety when using ART for critically ill individuals with HIV is a complex task that demands careful consideration of numerous factors. Despite the potential to overcome these barriers, many centers lack the resources to provide dedicated ARVSPs. The importance of such programs becomes evident due to the limited familiarity with antiretroviral medications beyond HIV clinicians. This lack of widespread knowledge among clinicians regarding ART can lead to challenges such as inappropriate dosing, drug interactions, and incorrect medication reconciliation, directly impacting patient well-being, potentially undermining the future efficacy of their ART, and contributing to poor outcomes. Recognizing these challenges, clinicians must proactively seek out resources and support to enhance their understanding of ART and optimize their management strategies for critically ill individuals with HIV. The complexities associated with using ART in this population necessitate a comprehensive approach to optimize patient outcomes and ensure patient safety. Moreover, additional research is required to enhance the management of ART in critically ill individuals with HIV and to assess the impact of rapidly initiating ART in this population.

## Notes

***Author contributions.*** D. B. Ch. and D. B. Cl.: conceptualization, writing—original draft, and writing—review and editing (leads). All authors: writing—original draft and writing—review and editing.

***Patient consent.*** This is a review article and does not contain any identifiable patient data or require patient consent.

***Disclaimer.*** The contents do not represent the views of the US Department of Veterans Affairs or the US government.
